# Metal-tolerant and siderophore producing *Pseudomonas fluorescence* and *Trichoderma* spp. improved the growth, biochemical features and yield attributes of chickpea by lowering Cd uptake

**DOI:** 10.1038/s41598-023-31330-3

**Published:** 2023-03-18

**Authors:** Asad Syed, Abdallah M. Elgorban, Ali H. Bahkali, Rajalakshmanan Eswaramoorthy, Rana Khalid Iqbal, Subhan Danish

**Affiliations:** 1 Department of Botany and Microbiology, College of Science, King Saud University, P.O. 2455, Riyadh, 11451 Saudi Arabia; 2 Department of Prosthodontics, Saveetha Dental College and Hospitals, Saveetha Institute of Medical and Technical Sciences (SIMATS), Chennai, 600 077 India; 3 Institute of Molecular Biology and Biotechnology, Bahauddin Zakariya University, Multan, Punjab Pakistan; 4 Department of Biology, University of Padova, Padua, Italy; 5 Department of Soil Science, Faculty of Agricultural Sciences and Technology, Bahauddin Zakariya University, Multan, Punjab Pakistan

**Keywords:** Plant sciences, Plant stress responses, Abiotic

## Abstract

Industrialization and human urbanization have led to an increase in heavy metal (HM) pollution which often cause negative/toxic effect on agricultural crops. The soil-HMs cannot be degraded biologically however, microbe-mediated detoxification of toxic HMs into lesser toxic forms are reported. Considering the potentiality of HMs-tolerant soil microbes in metal detoxification, *Pseudomonas fluorescence* PGPR-7 and *Trichoderma* sp. T-4 were recovered from HM-affected areas. Under both normal and cadmium stress, the ability of both microorganisms to produce different plant hormones and biologically active enzymes was examined. Strains PGPR-7 and T-4 tolerated cadmium (Cd) an up-to 1800 and 2000 µg mL^−1^, respectively, and produced various plant growth regulating substances (IAA, siderophore, ACC deaminase ammonia and HCN) in Cd-stressed condition. The growth promoting and metal detoxifying ability of both strains were evaluated (either singly/combined) by applying them in chickpea (*Cicer arietinum* L.) plants endogenously contaminated with different Cd levels (0–400 µg kg^−1^ soils). The higher Cd concentration (400 µg kg^−1^ soils) negatively influenced the plant parameters which, however, improved following single/combined inoculation of *P*. *fluorescence* PGPR-7 and *Trichoderma* sp. T-4. Both microbial strains increased the growth of Cd-treated chickpeas however, their combined inoculation (PGPR-7 + T-4) caused the most positive effect. For instance, 25 µg Cd Kg^−1^ + PGPR-7 + T4 treatment caused maximum increase in germination percentage (10%), root dry biomass (71.4%) and vigour index (33%), chl-a (38%), chl-b (41%) and carotenoid content (52%). Furthermore, combined inoculation of *P. fluorescence* PGPR-7 and *Trichoderma* sp. T-4 maximally decreased the proline, MDA content, POD and CAT activities by 50%, 43% and 62%, respectively following their application in 25 µg Cd kg^−1^ soils-treated chickpea. Additionally, microbial strains lowered the plant uptake of Cd. For example, Cd-uptake in root tissues was decreased by 42 and 34% when 25 µg Cd Kg^−1^- treated chickpea plants were inoculated with *P*. *fluorescence* PGPR-7, *Trichoderma* sp. T-4 and co-inoculation (PGPR-7 + T4) of both strains, respectively. Therefore, from the current observation, it is suggested that dual inoculation of metal tolerant *P*. *fluorescence* and *Trichoderma* sp. may potentially be used in detoxification and reclamation of metal-contaminated soils.

## Introduction

In today’s agriculture, heavy metals (HMs) pollution is regarded as the most serious environmental issue^1^. Due to various human and natural activity, ecosystems have become poisoned by heavy metals^2^. The following activities largely contributed to the release of heavy metals into the environment: metal mining and smelting, burning of fossil fuels, fertilizers, sewage sludge, pesticides, municipal wastes, pigment production, wasted batteries, and vehicle exhaust^3^. In moderation, HMs can be utilized as plant nutrients (Cu, Zn, Fe, Mn, Ni, and Co are necessary components); nevertheless, higher HMs concentration in soil often cause severe toxic effects on edible crops^4^ and thus, reduced the agricultural productivity^5^.

These HMs are taken excessively by plant roots and transported to shoots and other plant organs. These translocations affected the metabolism and caused the stunted growth in the shoots^6^. Also, HM pose a negative effect on morphological^7^, physiological^8^ and biochemical^9^ properties as well as nutritional uptake^10^ and symbiotic features^11^ of leguminous crops including chickpeas. In addition, HMs caused a significant reduction in soil microbial activity, soil fertility and yields losses^12^.

HMs present in soil cannot degraded, but can only be converted to organic compounds through a biological process^13^. Therefore, it is necessary to choose plants, which interact with bacteria and plant roots, and investigate adequate metal concentrations in soil to carry out successful phytoremediation operations. Metal-resistant microorganisms can aid in the removal of metals from soil. In this regard, various metal-tolerant plant growth promoting bacteria^14–16^ and fungi^17–19^ isolated from different metal-contaminated areas are reported to detoxify the HMs toxicity. By producing/ synthesizing various growth regulating active biomolecules, these metal tolerant microbes increase the growth and yield of metal-polluted crops. For instance, growth of cadmium supplemented *Sedum alfredii* (L.) were enhanced by inoculating Cd-tolerant and endophytic bacterium *Buttiauxella* sp*.* SaSR13. The strain further decreased the concentration of cadmium in root and shoot tissues^20^. Furthermore, *Bacillus* sp. ZC3-2-1, a Zn and Cd tolerating PGPR strain improved the growth and ionic homeostasis in rice plants treated separately with heavy metals. Additionally, bio-inoculation of this bacterium enhanced the phytoextraction of Cd and metal immobilization^14^. Compared to un-inoculated treatment, the application of Cd-resistant strain decreased the bioavailability of Zn (32%) and Cd (93.3%) in rice seedlings.

It is widely reported that fungal strains have the ability to tolerate high HMs ions concentrations, play a crucial role in metal detoxification/reclamation^21^ and plant growth promotion^22^ and which constitutes a biological barrier against the transfer to the shoot tissues. Till date, a number of metals and other stress tolerant plant growth promoting fungi (PGPF) have been reported as stress reliever and growth promoter. For example, in a study, Cr and As-tolerating fungus *Aspergillus welwitschiae* strain Bk was isolated and recorded to synthesize phytohormone, solubilized insoluble P and produced stressor metabolites^17^. Following inoculation, strain Bk enhanced the root and shoot length of metal-affected *Glycine max* (L.). Also, Bk supported the antioxidative responses of metal-supplemented *G. max* and increased the CAT (1.58-fold increase), AAO (6.75–7.94-fold increase), POD (1.37-fold) and 1,1-diphenyl-2-picrylhydrazyl; DPPH (1.42-fold increase) activities of plant. Furthermore, strain Bk lowered the uptake of chromium (Cr) and arsenic (As) by 50% in plants and thus, lowered the metal phytotoxicity. In another study, multi-metal tolerant *Aspergillus* *flvaus* strain CR-500 had multifarious biochemical, physiological and growth regulating properties. The strain CR-500 potentially removed the metals such as As (99%), Pb (97.7%) and Ni (73.1%) by the mean of bioaccumulation and surface adsorption mechanisms. *A. flavus* CR-500 also exhibited excellent multi-metal (As, Ni, Pb and Cr) removal potential from simulated wastewater (SWW) and Tannery wastewater (TWW) with a high amount of biomass production rate^23^. The metal tolerant microbes hold a complex mechanism to adapt to metal stresses (a) binding of Cd by the elements of the cell wall, as well as produced siderophores and exopolysaccharides, to prevent it from entering the cell; (2) metalloproteases sequester Cd intracellularly; (3) Cd is eliminated from the cell through efflux pumps; (4) antioxidants reduce the harmful effects of Cd. However, only a few reports are available on dual/combined inoculation effect of metal tolerant microbes. So, considering these, the current project was undertaken with following specific objectives: (i) assessment of tolerance of different among bacterial and fungal isolates (ii) evaluation of PGP activities of *P. fluorescence* PGPR-7 and *Trichoderma* sp. T4 strains under Cd-stressed condition (iii) single/combined inoculation effect of metal tolerant microbes on chickpea raised in pot soils contaminated with different level of Cd (iv) Effect of Cd-tolerant PGPR-7 and T4 strains (single and dual) on growth, dry biomass and photosynthetic pigments of Cd-treated chickpea (v) assessing the dual inoculations of *P. fluorescence* PGPR-7 and *Trichoderma* sp. T4 strains on antioxidative response of Cd-treated chickpea (vi) evaluating the uptake of metal in Cd-treated and microbial strain inoculated chickpea.

## Material and methods

### Isolation of microbial isolates

By using the serial dilution procedure, the rhizosphere microbial isolates were recovered from rhizosphere soils of vegetables field irrigated with industrial/wastewater. The isolation of soil bacteria was accomplished using the nutrient agar medium/king’s B medium. Aseptically adding one mL of soil suspension from dilutions (10^–5^ and 10^–6^) to sterile Petri plates with ten mL of sterile media and incubating for 48 h at 28 °C. Following incubation, distinct individual colonies with yellow, green, and white pigments were identified. The isolated cultures were obtained by picking up the individual colonies with sterile loops, transferring them to Nutrient agar media slants, and then storing them in a refrigerator at 40 °C for later use. In a similar fashion, fungal isolates *Trichoderma* were recovered using serial dilutions (10^–3^ and 10^–4^) that were aseptically added to sterile Petri plates that contained 20 mL of Potato Dextrose agar (PDA) medium and incubated 37 °C for 3 days. A total of 20 bacterial and 15 fungal isolates were recovered and assessed for their cultural and biochemical properties.

### In-vitro heavy metal testing of microbial isolates

In order to evaluate the heavy metal (HM) tolerant ability, recovered isolates were grown on Nutrient agar (NA; for bacterial isolates)^24^ and potato dextrose agar (PDA; for fungal isolates). Both of solid growth media were pre-supplemented with increasing doses (0–2400 µg mL^−1^) of salts of each chromium (Cr), cadmium (Cd). Lead (Pb), arsenic (As) and nickel (Ni)^25^. The freshly grown microbial (bacteria and fungi) isolates were spotted on metals-amended plates. Plates were incubated at 28 ± 2 °C for 48 h (for bacterial isolates) and 72 h (for fungal isolates) and observed for bacterial survivability. Bacterial and fungal isolates surviving the maximum level of metals were recorded as metal tolerant isolates. Of the total bacterial isolates (*N* = 20) and fungal isolates (*N* = 15), strain PGPR-7 and T-4 tolerated maximum concentrations of metals and therefore, selected for additional studies. All the isolates were morphologically and biochemically characterized.

### Molecular identification of bacterial isolate

Isolate PGPR-7 showed the maximum tolerance towards the HMs. Therefore, it was decided to identify the isolate to genus level. For this, DNA was isolated using a PGPR-7 culture that had been cultivated overnight. A microcentrifuge tube containing 2.0 ml of the bacterial suspension was filled with the pellet and centrifuged for two minutes at 10,000 × *g*. To obtain an adequate number of cells, the aforementioned procedure was carried out twice with 2.0 ml of bacterial suspension. In order to release the genomic DNA, 0.2 ml of protease was added after the cells had been rinsed with 0.9% saline. This process digested and removed the protein and cellular components from the cells. The centrifuged tubes were inverted five to six times and heated to 55 °C in a water bath for one hour. One hour later, 0.1 ml of the DNA Salt solution was added, and it was then centrifuged at 5,000 rpm for five minutes. The centrifuge tube was then repeatedly inverted to thoroughly mix the components before 0.8 ml of the precipitated solution was progressively added. The cells were washed with 70% ethanol. Dried and suspended in TE buffer, the obtained DNA was kept at 4 °C. A 8% agarose gel was used to verify the extracted DNA's purity. Utilizing universal primers, the 16S rRNA gene fragments were amplified. Through the use of a DNA sequencing service, amplified 16S rRNA gene fragments were purified. The 16S rRNA gene sequences were collected and submitted to BLAST, a programme that searches local alignments for nucleotide sequences that match those in reference sequences.

### Assessment of PGP traits of microbial strains under Cd stress

#### Evaluation of siderophore and indole-3-acetic acid production

For qualitative estimation of siderophore, FeCl_3_ test was performed by growing the microbial cultures in nutrient broth (NB) added with 0–400 µgCdmL^−1^^26^. Additionally, the production of siderophores were assessed using the previously described method^27^, which involved spot-inoculating of freshly grown cultures of strain PGPR-7 and T-4 onto chrome azurol S (CAS) agar plates that had been amended with different concentrations of Cd. The plates were examined for the development of a yellow to orange zone (halo), which indicates the release of siderophores, around bacterial growth after incubation. Additionally, employing Modi media, a quantitative assessment of siderophores was carried out. For this, 100 µL of microbial cell suspension was added to Modi medium that had been supplemented with 0–400, µg mL^−1^ of Cd. The inoculated tubes underwent a 5-day incubation period at 28 ± 2 °C. The salicylate (SA) and 2, 3-dihydroxybenzoic acid (DHBA), catechol type phenolates, were then measured in the supernatant after centrifuging the cultures at 6000 rpm.

By using the modified protocol of Mayer^28^, the IAA secretion by the metal-tolerant bacterial strain PGPR-7 and T-4 was measured. In order to conduct the test, 100 mL of overnight-grown culture was added to 25 mL of Luria Bertani (LB) broth that had already been supplemented with 100 µg of tryptophan per mL. It was then exposed to 0, 25, 50, 100, 200, and 400 µg Cd mL^−1^. After inoculation, the cultures underwent a 48-h incubation period at 28 ± 2 °C and 125 rpm in a shaking incubator. After incubation, 4 mL of Salkowsky reagent and 2–3 drops of orthophosphoric acid were added to the supernatant from a centrifugation of 2 mL of each treatment's culture at 10,000 *g* for 15 min. For the purpose of colour development, samples were left in the dark at 28 ± 2 °C for one hour. Using pure IAA as a reference, the amount of IAA released in the supernatant was calculated at 530 nm by measuring the absorbance of the pink colour that emerged during the reaction.

#### ACC Deaminase production

By cultivating and spot inoculating microbial cultures on Dworkin and Foster (DF) salts minimum medium enriched with 3.0 mM ACC as the main N source, it was possible to determine the ACC deaminase generated by the metal-tolerant strains PGPR-7 and T-4 in a qualitative manner. The negative and positive control plates were made with DF agar plates that contained (NH_4_)2SO_4_ (0.2% w/v) but no ACC. The plates underwent a 72-h incubation period at 28 ± 2 °C, during which time bacterial growth was observed. The modified approach of Penrose and Glick^29^ and Honma and Shimomura^30^ was applied to quantitatively estimate the ACC deaminase. By measuring the absorbance of samples against a standard curve of pure α-ketobutyrate, the amount of α-ketobutyrate released as a result of ACC breakdown was determined. The ACC deaminase activity was expressed as μ mol α-ketobutyrate mg^−1^ protein h^−1^.

#### Ammonia and hydrogen cyanide (HCN) production

Ammonia production activity of metal tolerant microbial strains (PGPR-7 and T-4) was assessed under metal-stressed condition. For the assay, freshly grown microbial strains were inoculated in peptone water amended with 0–400 μg Cd mL^−1^ incubated for 4 days at 28 ± 2 °C^31^. The colour changed from yellow to reddish brown, indicating a favourable response. Further, cyanogenic compound released by PGPR-7 and T-4 was detected following the method of Bakker and Schipper^32^. In order to do this, King's B agar plates containing 4.4 g L^−1^ glycine and supplemented with 0, 25, 50, 100, 200 and 400 µgmL^−1^ of Cd were spread plated. Filter paper discs coated in 0.5% picric acid and made with 2% sodium carbonate were used to seal the Petri plate lids. To prevent the bacterial strain's volatile HCN from escaping, parafilm was used to seal each experimental plate. The plates were then incubated for 4 days at 28 ± 2 °C. Positive response was said to have occurred when the yellow filter paper turned orange.

### Assessing the effect of metal tolerant microbial strains on chickpea raised in pot soils treated with Cd

#### Germination efficiency and growth parameters

For the purpose of evaluating how Cd treated chickpea plants react in the presence of single/combined inoculations of metal tolerant microbial strains (PGPR-7 and T-4). Uniform and healthy chickpea seeds were surface sterilised with 4% sodium hypochlorite (NaOCl) for 3 min. The seeds were then rinsed with 6–7 changes of sterile water. The seeds were sterilised before being immersed in metal-tolerant *P. fluorescence* PGPR-7 and *Trichoderm*a asp. T-4 (single/dual inoculation) using 1% guar gum powder as an adhesive to deliver about 10^8^ cells per seed. Additionally, the uncontaminated seedlings that were just steeped in sterile water served as a control. In 3.0 kg of non-sterilized alluvial sandy clay loam soil, the bio-primed and uninoculated seeds were planted in earthen pots. Two days before the seeds are sown, different concentrations (0, 25, 50, 100, 200 and 400 mg/kg soils) were added to soil. To replicate the quantities seen in the soil samples analysed, these Cd doses were used for the experimental trials. There were 23 treatments with three replications each and was as follows:

T1 = Control (un-inoculated), T2 = *P. fluorescence* PGPR-7, T3 = *Trichoderma* sp. T-4, T4 = 25 µg Cd kg^−1^ soil, T5 = 50 µg Cd kg^−1^ soil, T6 = 100 µg Cd kg^−1^ soil, T7 = 200 µg Cd kg^−1^ soil, T8 = 400 µg Cd kg^−1^ soil, T9 = 25 µg Cd kg^−1^ soil + *P. fluorescence* PGPR-7, T10 = 50 µg Cd kg^−1^ soil + *P. fluorescence* PGPR-7, T11 = 100 µg Cd kg^−1^ soil + *P. fluorescence* PGPR-7, T12 = 200 µg Cd kg^−1^ soil + *P. fluorescence* PGPR-7, T13 = 400 µg Cd kg^−1^ soil + *P. fluorescence* PGPR-7, T14 = 25 µg Cd kg^−1^ soil + *Trichoderma* sp. T-4, T15 = 50 µg Cd kg^−1^ soil + *Trichoderma* sp. T-4, T16 = 100 µg Cd kg^−1^ soil + *Trichoderma* sp. T-4, T17 = 200 µg Cd kg^−1^ soil + *Trichoderma* sp. T-4, T18 = 400 µg Cd kg^−1^ soil + *Trichoderma* sp. T-4, T19 = 25 µg Cd kg^−1^ soil + *P. fluorescence* PGPR-7 + *Trichoderma* sp. T-4, T20 = 50 µg Cd kg^−1^ soil + *P. fluorescence* PGPR-7 + *Trichoderma* sp. T-4, T21 = 100 µg Cd kg^−1^ soil + *P. fluorescence* PGPR-7 + *Trichoderma* sp. T-4, T22 = 200 µg Cd kg^−1^ soil + *P. fluorescence* PGPR-7 + *Trichoderma* sp. T-4, T23 = 400 µg Cd kg^−1^ soil + *P. fluorescence* PGPR-7 + *Trichoderma* sp. T-4.

A completely randomised block pattern was used to place the pots. Only two plants per container were kept alive until harvest fifteen days after the plants were thinned. The pots were maintained in open-air settings and periodically watered with tap water. To verify the accuracy and reproducibility of the results, the experiments were conducted repeatedly over the course of two years.

#### Photosynthetic pigments estimation

Fresh leaf samples (0.5 g) from each replicated Cd-treated and microbial inoculated treatment were homogenised in 10 mL of 80% methanol. The samples were kept at 4 °C overnight after being centrifuged for 10 min. at 12,000 rpm. Utilizing a UV visible spectrophotometer, the absorbance of the extract was measured at 663, 645, and 480 nm for the amounts of chlorophyll a, b, and total carotenoid^33,34^. The following equations were used to determine the chlorophyll and carotenoid content of plants;

$${\text{Chl}}\left( {\text{a}} \right)\left( {{\text{mg}}/{\text{g}}} \right) = \left[ {{12}.{7}\left( {{\text{OD663}}} \right) - {2}.{69}\left( {{\text{OD645}}} \right)} \right] \times {\text{V}}/{1}000 \times {\text{W}}$$

$${\text{Chl}}\left( {\text{b}} \right)\left( {{\text{mg}}/{\text{g}}} \right) = \left[ {{22}.{9}\left( {{\text{OD645}}} \right) - {4}.{68}\left( {{\text{OD663}}} \right)} \right] \times {\text{V}}/{1}000 \times {\text{W}}$$

$$\begin{gathered} {\text{V}} = {\text{Volume}}\;{\text{of}}\;{\text{the}}\;{\text{extract}}\;\left( {{\text{mL}}} \right), \hfill \\ {\text{W}} = {\text{Weight}}\;{\text{of}}\;{\text{fresh}}\;{\text{leaf}}\;{\text{tissue}}\;\left( {\text{g}} \right) \hfill \\ {\text{Carotenoids}}\;\left( {{\text{mg}}/{\text{g}}} \right):\;\left( {{\text{OD at 48}}0} \right) \times {4} \hfill \\ \end{gathered}$$

#### Estimation of proline and MDA content

The method of Bates et al.^35^ and previously done by Shahid et al.^36^ was followed to assess the proline contents in the fresh leaves of plants that had been either treated with increasing Cd concentrations and inoculated with metal tolerant microbial strains. In a nutshell, 5.0 mL of 3% (w/v) aqueous sulfosalicylic acid were used to homogenise 1.0 g of leaf samples. The homogenates were filtered through Whatman No. 2 filter paper and centrifuged at 8000 rpm for 20 min before being treated with acid ninhydrin (2.0 mL) and glacial acetic acid (2.0 mL) at 80 °C for one hour before being stopped in an ice bath. The colourful complex that formed was extracted with 4 mL of toluene, and absorbances at 520 nm were measured.

An evaluation of the MDA concentration was used to estimate the amount of lipid peroxidation. In this instance, 1.0 g of fresh tissue was homogenised in 10 mL of 10% (w/v) TCA and centrifuged for 10 min at 10,000 rpm. The supernatant was added to a 10% trichloroacetic acid (TCA) solution that included 0.5% of 2-thiobarbituric acid (TBA) and 1% polyvinylpyrrolidone (PVP) in an equal proportion. The sample was incubated for 30 min at 95 °C, immediately cooled in an ice bath, then centrifuged for 15 min at 10,000 rpm. In order to account for nonspecific absorbance, the absorbance was measured at 532 nm and corrected at 600 nm. Using an extinction value of 155 mM^−1^ cm^−1^ as M (MDA)g^−1^ FW, the concentration of MDA was determined.

#### Antioxidant enzymatic activities

1.0 g of fresh root and leaf samples were pulverised in liquid nitrogen with a mortar and pestle. The ground materials were homogenised on ice in 10 ml of a solution containing 50 mmol/l potassium phosphate buffer and 1% (w/v) PVP (pH 7.8), and the mixture was then maintained at 4 °C for 10 min. Filtering and centrifuging the homogenates at 4000 × *g* for 15 min. at 4 °C. After then, the antioxidant enzyme activity of the supernatant was evaluated. On a blank that didn't include any protein extract, each enzyme's activity was tested.

Extracts for the POX enzyme was made by combining 250 mg of crushed plant material with 1.2 mL of a 50 mM Tris–HCl buffer (pH 7.3) containing 0.5 M of CaCl_2_ and 5.0 mM of -mercaptoethanol, and then incubating the mixture at 4 °C for an hour. A crude extract of POX was made from the supernatant after the homogenate underwent centrifugation (14,000 × *g* 4 °C, 45 min). The homogenate was centrifuged (14,000 × *g,* 4 °C, 45 min) to produce a supernatant, and the supernatant was used to create a crude extract of POX^37–39^.

Following the breakdown of H_2_O_2_, the catalase (CAT) (EC 1.11.1.6) activity was measured as a reduction in absorbance at 240 nm for 1 min. The reaction mixture (total volume: 3 ml) contained the following ingredients: 20 µL of crude enzyme extract in 50 mM phosphate buffer (pH 7.0), 15 mM H_2_O_2_, and 25 °C. A blank solution of potassium phosphate buffer (50 mmol/L, pH 7.0) was used to measure the decrease in H_2_O_2_ absorbance during the 2 min experiment.

#### Assessment of inoculation effect of metal tolerant microbes on yield attributes and nutrient uptake in chickpea

At harvest, the grain attributes (seed number, seed weight and seed protein) were estimated in Cd-treated and microbial inoculated plants. The nutritional (nitrogen; N and phosphorous; P) content accumulated in metal treated and microbe-inoculated chickpea seeds were determined following the method of Jackson^40^ and Iswaran and Marwah^41^, respectively.

#### Inoculation effect of tolerant strains on Cd uptake

The uptake of Cd in dried organs (root and shoot) of bio-inoculated and cadmium-treated chickpea was analysed^42^. In order to do this, 100 mg of dried plant samples were digested separately in a mixture (ratio of 4:1) of HNO_3_ (nitric acid) and HClO_4_ (perchloric acid). After being digested, the residual suspensions were filtered through Whatman no. 2 filter paper and the volume was made up to 100 mL with double distilled water (ddH_2_O) and the content of Cd in each sample was determined by atomic absorption spectrophotometer (AAS) employing CRM standards (JT Baker, BAKER INSTRA-ANALYZED Reagent Grade for Trace Metal Analysis) Cd [1000 μg mL^−1^ (0.10% w/v)].

### Statistical analysis

Each *in-vitro* and *in-vivo* experiments were designed/arranged and conducted in three replicates (*n* = 3) and arranged in a completely randomized design (CRD) factorial. The database for the measured parameters and findings was made using Excel sheets. To examine the gathered data, Statistics 8.1 was used to perform an analysis of variance. Results were statistically analysed using Minitab-17.1 software and One-way ANOVA. Additionally, Duncan's Multiple Range Test (DMRT) was performed on data at a 5% confidence level to compare the treatment means.

## Results and discussion

### Isolation of microbial culture, tolerance of heavy metals and molecular identification

**Figure 1:**
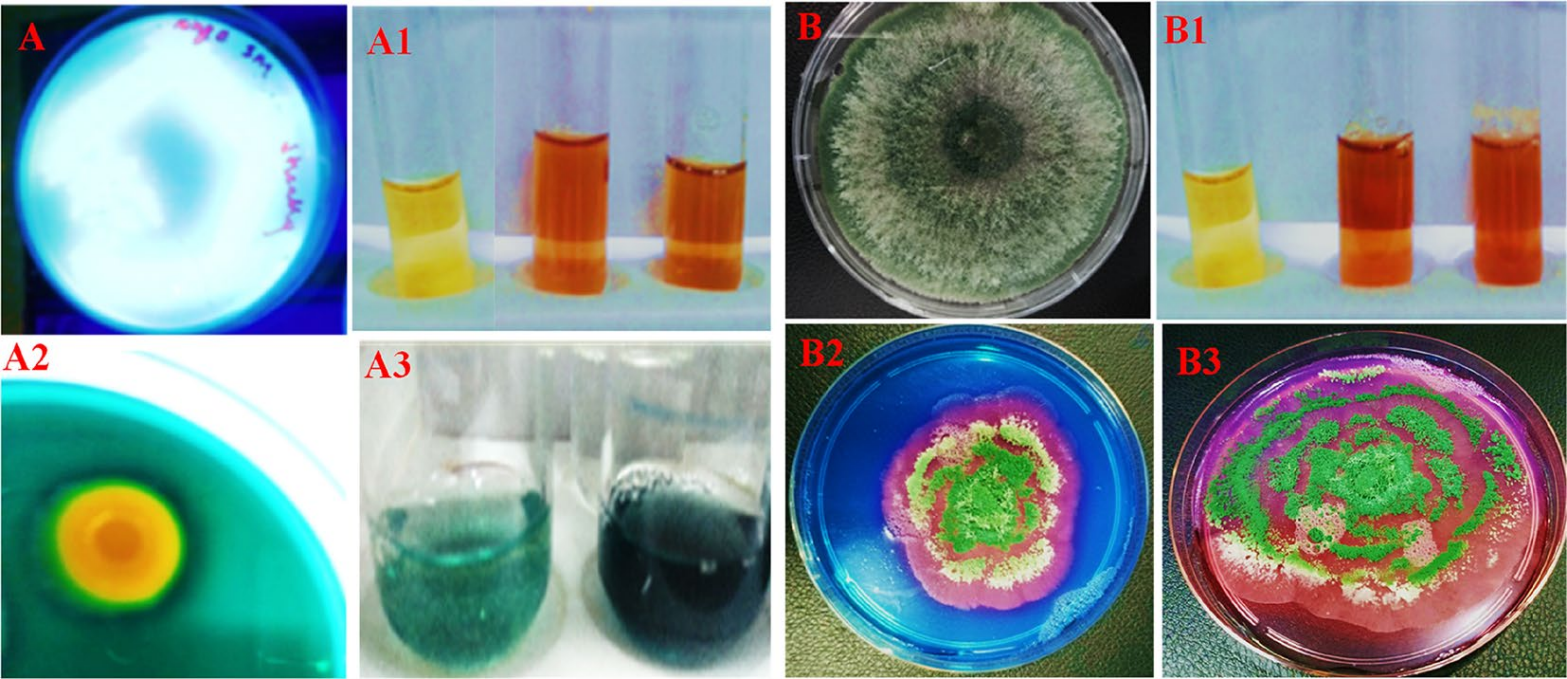
*Pseudomonas* PGPR-7 strain under UV light showing fluorescent (**A**). Ferric chloride (FeCl_3_) test indicating the production of siderophore (**A1**). Zone of halo formed by PGPR-7 strain on CAS gar plate (**A2**), and quantification of phenolate siderophore (**A3**). *Trichoderma* culture on PDA plate (**B**), FeCl_3_ test for siderophore production (**B1**). Panels **B2** and **B3** depicting the siderophore production ability of *Trichoderma* sp. T4.

**Figure 2:**
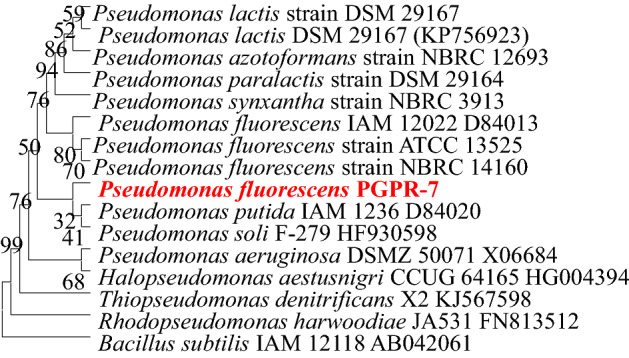
Neighbor-joined phylogenetic tree of *Pseudomonas fluorescence* PGPR-7. The tree was constructed based on 16S rRNA partial gene sequence and closely related phylogenetic species (type cultures) derived using the NCBI BLAST search tool. Sequences were aligned using Clustal W sequence alignment tool in MEGA 7.0 software. The GenBank accession numbers of isolates and closely related species are presented in brackets.

Globally, heavy metal contamination is a significant obstacle to the sustainability of the food supply since it frequently affects plant growth, nutrient intake, photosynthesis, and other physiological functions. In order to re-solve this issue, we tried to isolate HMs tolerant microbes (bacteria and fungi) that may be utilised as a microbial resource to enhance the performance and yield of crops in metal-stressed conditions. Here, a total of 20 rhizosphere bacteria and 15 rhizosphere fungi were collected and assessed for their cultural and biochemical features. Additionally, the ability of each microbial isolate to produce/synthesize active biomolecules that promote plant development was evaluated. Both of the strains were found as strong siderophore producer and a considerable amount of phenolate siderophore was quantified (Fig. 1). Furthermore, all the recovered bacterial and fungal isolates exhibited a variable tolerance to each metal. Among the bacterial isolates, strain PGPR-7 tolerated a maximum concentration of Cd (800 µg mL^−1^), Cr (1000 µg mL^−1^), Pb (800 µg mL^−1^) and Ni (1200 µg mL^−1^). Moreover, *Trichoderma* T-4 isolate among fungi tolerated metals and survived even in the presence of 1000, 1200, 800 and 1400 µgmL^−1^ each of Cd, Cr, Pb and Ni, respectively (Table 1). The bacterial strain PGPR-7 was molecularly characterized using 16S rRNA sequencing and identified as *Pseudomonas fluorescence*. A phylogenetic tree was constructed based on the similar sequences obtained from NCBI data base (Fig. 2).

**Table 1:** Tolerance of heavy metals (HMs) to isolated PGPR and Fungi.

Microbes	Heavy Metals (HMs) µg/mL			
	Cadmium (Cd)	Chromium (Cr)	Lead (Pb)	Nickle (Ni)
PGPR-1	200	800	400	1200
PGPR-2	400	800	200	1000
PGPR-3	100	400	50	800
PGPR-4	400	800	400	1000
PGPR-5	200	400	800	1200
PGPR-6	400	800	200	1000
PGPR-7	800	1000	800	1200
PGPR-8	100	200	400	1000
PGPR-9	50	100	200	800
PGPR-10	200	400	400	800
PGPR-11	400	800	400	200
PGPR-12	200	100	100	400
PGPR-13	50	100	200	400
PGPR-14	50	100	200	200
PGPR-15	25	100	400	1000
PGPR-16	50	100	200	200
PGPR-17	100	100	100	400
PGPR-18	25	50	50	400
PGPR-19	50	100	200	200
PGPR-20	100	100	400	800
T-1	400	1000	800	1000
T-2	200	200	400	1000
T-3	400	100	200	1200
T-4	1000	1200	800	1400
T-5	400	400	600	600
T-6	200	400	800	400
T-7	400	100	800	400
T-8	400	200	800	200
T-9	800	100	400	800
T-10	400	400	400	800
T-11	100	800	400	400
T-12	200	200	200	400
T-13	600	400	200	200
T-14	800	400	100	200
T-15	400	100	100	800

The capacity of this strain to withstand high metal concentrations is an intriguing feature since such microbes being may be utilised as an inoculant to increase crop productivity even when exposed to metal stress. Like the current observation, soil isolates recovered from metal polluted soil have been reported to displayed a dissimilar heavy metal tolerance profile^24^. Similarly, PGPR strain like *Bacillus* sp., *Cupriavidus* sp., *Cupriavidus* sp., *Fulvimonas soli* and *Novosphingobium* sp., isolated from rhizosphere soil tolerated heavy metal concentrations^43^. In yet another study, Fahsi and co-workers^44^ reported that *Enterobacter hormaechei* J-146 could withstand up to 1.5 mg L^−1^ of Cu/Cd, while *B. halotolerans* J-143 could tolerated up to 1.5 mg L^−1^ of Ni. Furthermore, *P. frederiksbergensis* J-158, was found to be the least metal tolerating microbe. Furthermore, numerous metal tolerant fungi like *Alternaria* sp., *Bipolaris zeicola*^45^, *Trichoderma brevicompactum*^25^ and *Aspergillus welwitschiae*^17^ have been reported for their crucial role in metal-clean up and crop enhancement. Global discussion and investigation surround the reason why microbial populations vary so greatly in their capacity to withstand such dangerous contaminants like heavy metals. According to some research, certain heavy metals cause different microorganisms to behave differently when it comes to metal tolerance. The reason behind could be (a) the variations in nutritional elements (macro- and micro) that are present in soils and growing medium that support bacterial development (b) dissimilarities in genetic constituents of microbial strains and (c) growth conditions/environmental variables affecting the growth of microorganisms^46^. Additionally, metal-tolerant microbes, which might be used as a bioremediation tool, have the ability to complex metals inside of cells, expel metal ions outside of cells, and enzymatically degrade/transform specific metal ions. These are some of the methods through which metal-tolerant bacteria can prevent the negative effects of metals on soil and plant health, hence reducing the risks to human health, whether they are used separately or in tandem. The metal tolerant microbial strains PGPR-7 and T-4 were subsequently characterized and molecularly identified to species level. Strain PGPR-7 was a Gram-positive, rod-shaped bacteria that fluoresced green on nutrient agar plates. The morphological and biochemical features of the plant were evaluated. According to its biochemical and cultural characteristics, PGPR-7 belonged to the genus *Pseudomonas*, and the 16S rRNA gene sequencing revealed that it was *Pseudomonas* at the species level. The 16S rRNA nucleotide sequence of strain PGPR-7 was deposited to GenBank. The bacterium PGPR-7 was identified as *Pseudomonas fluorescence* after a similarity search using the BLASTn programme showed that the two were closely related (99.64% sequence identical). Given that the study of the 16S rRNA gene sequence is a widely used technique for identifying bacterial strains, a phylogenetic tree was constructed using MEGA 7.0 software.

### Growth regulators of PGPR-7 and T-4 strains under Cd stress

#### Siderophores and IAA

The metabolically important class of substances called siderophores is released by soil bacteria and fungi, and they chelate iron from the medium to prevent plant pathogens from absorbing it^47,48^. So, siderophores indirectly support plant development and survival in metal-polluted soils by reducing the number of pathogens^49,50^. The microbial strain PGPR-7 and T4 employed in this investigation was capable of producing an 19.0 mm and 25.0 mm zone on CAS agar medium, respectively and synthesising siderophores in the absence of metals (Table 2). When cultured in liquid medium as opposed to solid medium, both microbial strains continued to produce siderophores even when exposed to Cd stress. PGPR-7 produced 9.23 μg mL^−1^ SA and 5.51 μg mL^−1^ 2, 3-DHBA when cultured in the presence of 25 μg Cd mL^−1^, which were 3% (Salicylate) and 2% (2,3-DHBA) reduced over untreated (0 μg Cd mL^−1^) control. Similarly, Cd at 200 μg mL^−1^ decreased the fungal siderophore production of by 14.4% (2,3-DHBA) and 211.6% (SA) while comparing control (Table 2). It's interesting to note that both metal tolerant microbial strains continued to produce siderophores at all concentrations of Cd, despite the fact that the levels were relatively insufficient at higher metal doses. Like our investigation, various metal-resistant and siderophore synthesizing soil bacteria such as *Bacillus aerophilus*^51^, *Pseudomonas putida*^52^, *Enterobacter* sp.^53^ and fungi like *Piriformospora indica*^54^, *Trichoderma virens*^55^, *Aspergillus aculeatus*^56^ are reported to augment the growth of different crops endogenously polluted with heavy metals.

**Table 2:** Plant growth regulating activities of HMs tolerant microbes under Cd stress.

Microbes	Cd concentration (µg/mL)	Siderophore				ACC deaminase	IAA (µg/mL)	HCN	NH_3_
		FeCl_3_ test	Size of halo zone (mm)	SA (µg/mL)	2, 3-DHBA (µg/mL)				
*Pseudomonas fluorescence* PGPR-7	0	+ + +	19 (c)	9.23 (c)	5.51 (c)	13.9 (d)	123.1 (a)	+ +	+ +
	25	+ +	19 (c)	9.0 (c)	5.02 (d)	13.3 (d)	120.1 (a)	+	+ +
	50	+ +	18 (c)	8.12 (d)	4.21 (d)	12.6 (e)	115.6 (b)	+	+
	100	+	15 (d)	7.29 (e)	4.01 (e)	11.8 (e)	109.2 (b)	−	+
	200	+	15 (d)	5.77 (f)	3.09 (f)	10.5 (f)	102.6 (c)	−	+
Mean	–	−	17.2	7.88	4.36	12.4	113.9	−	−
*Trichoderma* sp. T-4	0	+ +	25 (a)	13.4 (a)	8.44 (a)	21.4 (a)	72.3 (d)	+ +	+ +
	25	+ +	24 (a)	12.0 (a)	7.13 (b)	20.8 (a)	70.5 (d)	+	+ +
	50	+	22 (b)	11.7 (b)	6.09 (c)	21.0 (a)	67.8 (e)	+	+ +
	100	+	20 (b)	12.3 (b)	7.41 (b)	19.8 (b)	71.3 (d)	−	+
	200	+	18 (c)	10.5 (c)	7.22 (b)	17.2 (c)	56.3 (f)	−	−
Mean	–	−	21.8	11.9	7.2	20.0	67.6	−	−

Value indicates the means (mean ± S.D) of three independent replicates. Means followed by similar alphabets are significantly different from each other according to Duncan’s multiple range test (DMRT). Here, Cd = cadmium, µg/mL = micrograms per milliliters, FeCl_3_ = ferric chloride, SA = salicylic acid, DHBA = dihydroxy benzoic acid, ACC = 1-amino cyclopropane decarboxylate, IAA = indole acetic acid, HCN = hydrogen cyanide, NH_3_ = ammonia.

Even when cultivated in soils with metal contamination, the IAA generated by soil microbiota has been shown to affect the plant growth by speeding up cell division and enlargement as well as causing root elongation and apical dominance in plants. So, considering this significance, the amount of IAA synthesized by *P*. *fluorescence* PGPR-7 and *Trichoderma* sp. T-4 under Cd-stressed condition was assessed. IAA was synthesized at its highest levels by strain PGPR-7 (123.1 µg mL^−1^) and T-4 (72.3 µg mL^−1^) when grown in media free of Cd among other plant growth promoters which, however, decreased by 17 and 22%, respectively when grown in LB broth was treated with 200 µg CdmL^−1^ (Table 2). Comparing the effects of different Cd concentrations on IAA generation by microbial strains, the highest measured concentration had the deadliest effect. Therefore, it was clear from the current results that both microbial strains maintained the plants' release of IAA into the outside environment even when growing at greater concentrations of Cd. In contrast to normal metal-free conditions, the amount of IAA that diffused externally was worse under metal stress. Similar to the tested bacterial and fungal strains, it has been discovered that numerous bacteria^44,57,58^ and fungi^17,59^ inhabiting rhizospheres produce indole acetic acid and have a favourable impact on a number of crucial physiological processes in plants. Therefore, this metal tolerant strain's capacity to promote IAA in addition to its ability to alleviate heavy metal stress could be a compelling choice for encouraging wheat plant growth even in metal-stressed conditions.

#### HCN and ammonia

A significant element that indirectly promotes plant development by stopping the formation of pathogens is hydrogen cyanide (HCN), a volatile secondary metabolite generated by numerous bacteria. Numerous soil bacterial and fungal species can produce HCN even when under metal pressure, which could greatly aid in boosting crop output in metal contaminated soil. Here, both microbial strains (PGPR-7 and T-4) could secrete HCN in metal free as well metal-stressed condition. Our findings are supported by the fact that various bacterial taxa, including *Bacillus* sp., and *Pseudomonas* sp. has a strong historical record of HCN production under stressed environment. Similarly, different doses of Cd had no discernible impact on ammonia produced by both of the microbial strains. However, the higher concentration of Cd lowered the production of ammonia, this could be seen from the way the colour of the resulting peptone water changed after being incubated. And, Cd at 200 μg mL^−1^ completely reduced the NH_3_ production activity of *Trichoderma* sp. T-4.

#### ACC deaminase

Numerous soil microbes including bacteria^60–62^ and fungi^63,64^, release the ACC deaminase enzyme, which is crucial in reducing ethylene levels in higher plants and promoting plant development even in stressful conditions. In light of this, the ability of both microbial strains to synthesise ACC deaminase was examined under metal stressed situations. The *P*. *fluorescence* PGPR-7 and *Trichoderma* sp. T-4 could produce 13.9 and 21.4 µmol α-ketobutyrate mg/g protein/h ACCD under metal-free environment. Even when different level of Cd was present, PGPR-7 and T-4 strains demonstrated ACC deaminase activity; however, this activity continually decreased as Cd concentrations increased. Among the Cd concentrations, 200 µg mL^−1^ induced a significant reduction (24%) fall in ACC deaminase activity of PGPR-7 strain in comparison to the untreated control (Table 2). It also showed the greatest reduction in α-ketobutyrate concentration. The concentration of α-ketobutyrate, in contrast, decreased to 17.2 mol mg/g protein/h in the presence of 200 µgCdmL^−1^ as opposed to very high production of ACCD (21.4 mol mg/g protein/h) by *Trichoderma* sp. T-4 (Table 2). It's interesting to note that the ability of both microbial strains to synthesise α-ketobutyrate in the presence of cadmium revealed that ACC deaminase coding genes of both soil microbes were expressed under metal-stressed condition. Other researchers have also noted that this specific property of microbial strains producing ACC deaminase enzyme even while under metal stress may be advantageous for promoting the development and subsequent production of agronomic crops even in metal-contaminated soils.

### Single/dual inoculation effect of *P*. *fluorescence* PGPR-7 and *Trichoderma* sp. T-4 on chickpea raised in Cd-stressed soil

#### Germination, biometric characteristics and photosynthetic molecules

The germination and growth of chickpea plants inoculated with metal tolerant microbial strains and varying level of Cd was variable. Increasing cadmium doses decreased the chickpea germination. For instance, Cd at 400 µg kg^−1^ reduced the germination of chickpea seedlings by 33.3% over untreated control. In contrast, while using metal tolerant microbes as bio-inoculant, PGPR-7 and *Trichoderma* sp. T-4 strains increased the germination efficiency of 25 µg Cd kg^−1^ soil treated chickpea by 10% and 11% compared to un-inoculated but treated with identical Cd concertation (Table 3). The dual inoculation caused the maximum enhancement in germination rate (10% increase). As well, root and shoot length of metal-treated plant declined with increasing Cd concentration which, however, improved following metal-tolerant microbial inoculation in polluted soils. As an example, root length of 25 µg Cd kg^−1^ soil treated plant were increased by 25% and 21% after soil inoculation of PGPR-7, *Trichoderma* sp. T-4 and PGPR-7 + *Trichoderma* sp. T-4, respectively, over un-inoculated control (Table 3). The plant length of chickpea of PGPR-7, *Trichoderma* sp. T4 and PGPR-7 + *Trichoderma* sp. inoculated and 25 µg Cd kg^−1^ soil treated plants were enhanced by 26.5%, 23% and 34%, respectively (Table 3). Likewise, metal tolerating PGPR strain *Bacillus anthracis* PM21 increased the germination efficiency and growth parameters of *Sesbania sesban* (L.) grown in soil treated with increasing concentrations of heavy metal^65^.

**Table 3:** Single and combined inoculation impact of metal tolerant *Pseudomonas fluorescence* PGPR-7 and *Trichoderma* sp. T-4 on biological parameters of chickpea raised in Cd affected soils.

Bio-inoculants	Cd concentrations (µgkg^−1^ soil)	Plant Length (cm)			Dry biomass (gm)			Percent germination (%)	Vigor index (VI)
		Root	Shoot	Total	Root	Shoot	Total		
UC	0	18.5 ± 2.4	28.9 ± 1.7	47.7 ± 4.1	0.6 ± 0.03	1.2 ± 0.21	1.8 ± 0.24	90	3456 ± 23
	25	17.0 ± 1.5	28.0 ± 2.1	45.0 ± 3.6	0.53 ± 0.1	1.1 ± 0.17	1.63 ± 0.27	90	3143 ± 18
	50	15.0 ± 1.1	26.0 ± 1.4	41.0 ± 2.5	0.48 ± 0.03	1.0 ± 0.0	1.48 ± 0.03	90	2678 ± 231
	100	12.0 ± 0.7	23.2 ± 0.8	35.2 ± 1.5	0.34 ± 0.0	0.88 ± 0.01	1.22 ± 0.01	85	2412 ± 45
	200	10.1 ± 0.2	20.0 ± 0.5	30.1 ± 0.7	0.21 ± 0.0	0.56 ± 0.03	0.77 ± 0.03	80	2001 ± 56
	400	6.5 ± 00	14.0 ± 1.2	20.5 ± 1.2	0.1 ± 0.0	0.37 ± 0.0	0.47 ± 0.0	60	1564 ± 87
Mean	–	13.1	23.3	36.5	0.37	0.85	1.22	82.5	2542
*P. fluorescence* PGPR-7	0	24.2 ± 2.3	40.2 ± 3.6	64.4 ± 5.9	1.10 ± 0.2	1.8 ± 0.12	2.9 ± 0.32	100	4321 ± 98
	25	22.5 ± 0.7	35.3 ± 2.5	57.8 ± 3.2	0.89 ± 0.12	1.65 ± 0.01	2.54 ± 0.13	100	3786 ± 56.4
	50	21.4 ± 0.23	35.0 ± 1.3	56.4 ± 1.53	0.69 ± 0.07	1.4 ± 0.04	2.0 ± 0.11	95	3219 ± 88
	100	17.4 ± 0.5	31.2 ± 1.0	48.6 ± 1.5	0.56 ± 0.03	1.0 ± 0.0	1.56 ± 0.03	90	2812 ± 79.0
	200	14.0 ± 0.21	25.2 ± 0.0	39.2 ± 0.2	0.42 ± 0.05	0.76 ± 0.0	1.16 ± 0.05	90	2431 ± 23.3
	400	8.5 ± 0.6	17.2 ± 0.76	25.7 ± 1.36	0.18 ± 0.0	0.53 ± 0.0	0.71 ± 0.0	80	1800 ± 17.8
Mean	–	18.0	31.01	48.6	0.64	1.19	1.81	92.5	3061
*Trichoderma* sp. T-4	0	23.1 ± 1.2	38.4 ± 1.6	61.5 ± 2.8	1.04 ± 0.31	1.76 ± 0.2	2.8 ± 0.51	100	4121 ± 69.2
	25	22.0 ± 1.6	36.0 ± 0.34	58.0 ± 1.94	1.0 ± 0.10	1.52 ± 0.016	2.52 ± 0.11	100	3890 ± 78.2
	50	19.6 ± 0.8	33.1 ± 0.8	52.7 ± 1.6	0.63 ± 0.09	1.37 ± 0.02	2.0 ± 0.1	95	3456 ± 89.0
	100	18.0 ± 0.32	29.0 ± 1.5	47.0 ± 1.82	0.59 ± 0.11	1.12 ± 0.01	1.71 ± 0.12	90	3100 ± 34.7
	200	13.1 ± 0.12	23.2 ± 2.4	36.3 ± 2.52	0.40 ± 0.01	0.78 ± 0.02	1.18 ± 0.03	90	2521 ± 80.1
	400	7.0 ± 0.0	15.6 ± 0.2	22.6 ± 0.2	0.15 ± 0.0	0.47 ± 0.0	0.62 ± 0.0	80	1987 ± 45
Mean	–	17.1	30.8	46.3	0.63	1.17	1.79	92.5	3179
*P. fluorescence* PGPR-7 + *Trichoderma* sp. T-4	0	29.6 ± 2.3	42.2 ± 1.8	71.8 ± 4.1	2.1 ± 0.5	2.76 ± 0.4	4.86 ± 0.9	100	5213 ± 33.3
	25	27.3 ± 1.8	42.1 ± 0.5	69.4 ± 2.3	2.0 ± 0.23	2.45 ± 0.12	4.45 ± 0.35	100	4891 ± 57.8
	50	25.2 ± 1.4	38.7 ± 2.1	64.0 ± 3.5	1.89 ± 0.12	2.21 ± 0.06	4.1 ± 0.18	100	4210 ± 87.9
	100	23.1 ± 2.1	34.2 ± 0.8	57.3 ± 3.0	1.56 ± 0.07	2.01 ± 0.14	3.56 ± 0.16	95	4100 ± 77.8
	200	21.3 ± 0.71	30.5 ± 0.12	51.3 ± 0.83	1.23 ± 0.04	1.73 ± 0.11	2.99 ± 0.15	95	3711 ± 45.6
	400	10.5 ± 0.24	23.1 ± 0.43	33.6 ± 0.67	1.03 ± 0.01	1.62 ± 0.07	2.65 ± 0.08	95	3112 ± 33.0
Mean	–	22.8	35.1	57.9	1.63	2.12	3.77	97.5	4206

Each value is a mean of three replicates where each replicate constituted three plants/pot. Mean values are significantly at *P* ≤ 0.05. Means followed by similar alphabets are different from each other according to DMRT test. Here, Cd = cadmium, µg/kg = micrograms per kilograms, VI = vigor index, % = percentage.

Additionally, dry biomass of Cd-treated plant was reduced in a concentration-dependent way. when chickpea plants were grown in the presence of 400 µg Cd kg^−1^ as opposed to the uninoculated control, the dry matter accumulation in the roots and shoots were reduced by 83.3% and 69%, respectively (Table 3). An earlier study conducted by Imtiaz et al. (2018) supported our findings noted a comparable drop in a number of biological indices in chickpea plants cultivated in the presence of different concentrations of vanadium (Vd). Metal-tolerant microbes detoxify the metal toxicity and enhanced the root and shoot dry biomass of plant. It's interesting to note that adding metal tolerant PGPR-7 to the experimental soils treated with 25 µg Cd kg^−1^ increased the root and shoot dry biomass by 52% and 37%, respectively, over non-inoculated control (Table 3). Like the current observation, a study found that *Zea mays* (L.) plants cultivated in the presence of increasing Pb concentrations displayed a concentration-dependent drop in germination rate, length of plant organs, fresh and dry biomass, and total protein content^66^. Additionally, a notable improvement in the development indices, such as the rate of germination, and growth parameters was recorded when dual inoculation were subjected to chickpeas grown in Cd stressed soil. These results imply that the addition of metal tolerant microbes to the culture reduced/eliminated the toxicity of heavy metals because of its capacity to detoxify metals, and as a result, enhanced a number of parameters of wheat plants grown in metal-treated soils.

Furthermore, in the presence of 400 µg Cd kg^−1^ soil, the Chl a, chl b and carotenoid content accumulated in fresh foliage of chickpea plants were significantly (*p* ≤ 0.05) reduced by 48, 56 and 67%, respectively. However, the leaves of inoculated plants showed more chlorophyll than the uninoculated one. Previous studies have found that under heavy metal stress circumstances, plant chlorophyll concentrations are lowered, photosynthetic pigment concentrations fluctuate, and gas exchange factors are inhibited. This drop in plant chlorophyll content may be caused by ROS interference, chlorophyll activation, modifications to important thylakoid membrane components, their manifestation mechanism, and modifications to the ultrastructure of proteins and DNA.

**Figure 3:**
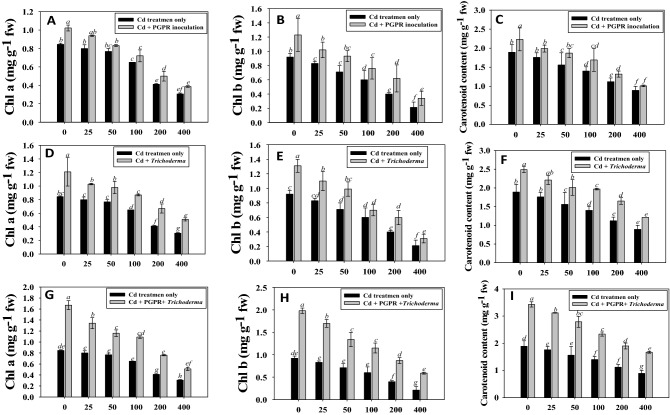
Single inoculation effect of PGPR (**A**–**C**), *Trichoderma* spp. (**D**–**F**) and combined inoculation of PGPR + *Trichoderma* sp. (**G**–**I**) on chl a, chl b and carotenoid content of fresh foliage detached from chickpea plants raised in soil affected with 0, 25, 50, 100, 200 and 400 μgCdmL^−1^. Each bar diagram represents the mean of three replicates (*n* = 3). Corresponding error bars represent standard deviation (S.D.) of replicates. Different letters represent that mean values are significantly different according to DMRT test.

Contrarily, a 34, 29 and 41% increase in chl a, chl b and carotenoid content was recorded when strain PGPR-7 was applied to chickpea plants raised in soil treated with 25 µg Cd kg^−1^ soil, respectively (Fig. 3). In addition, *Trichoderma* sp. T-4 inoculation enhanced the chlorophyll a, chl b and carotenoid content by 28, 32 and 38% even at 100 µg Cd kg^−1^ soil and successfully bioremediated experimental soil that had been exposed to metal. Similar research showing the disruption in ability of plant to photosynthesize when *Davidia involucrata* plants were exposed to increasing doses of Cd and Pb stress has been reported^67^. In contrast, research of plant inoculated with metal-tolerant PGPR strain and cultivated under Cd stress discovered a significant increase in leaf photosynthetic pigments and other important growth indices of *Glycine max* and *Lolium multiflorum*^68^.

#### Response of metal tolerant strains to antioxidant enzymes of Cd-treated chickpea

**Figure 4:**
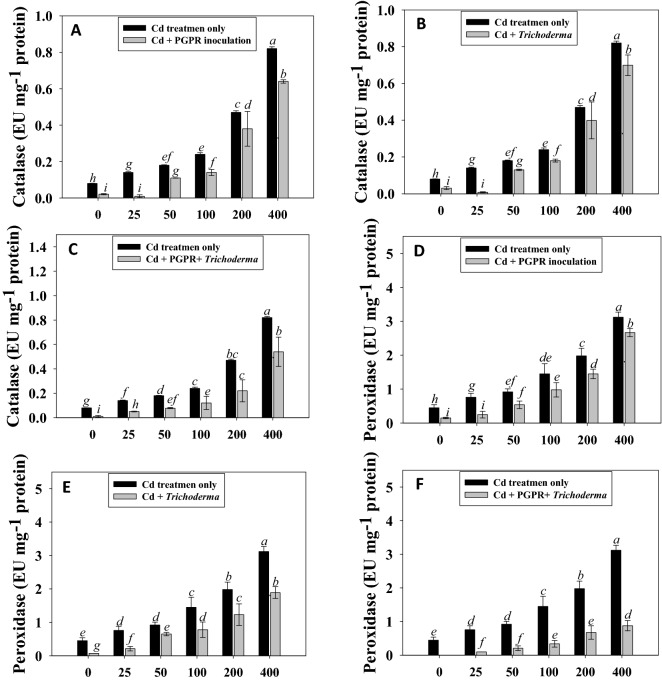
Antioxidant enzymes; catalase (**A**–**C**) and peroxidase (**D**–**F**) activities in cadmium supplemented and inoculated (either single/combined) with PGPR-7, *Trichoderma* sp. T4 strain. Each bar diagram represents the mean of three replicates (*n* = 3). Corresponding error bars represent standard deviation (S. D.) of replicates. Different letters represent that mean values are significantly different according to DMRT test.

When inoculated/uninoculated chickpea plants were grown in the presence of 0–400 µg/kg doses of Cd, a dose-dependent increase in the antioxidant enzyme activity of the foliage was observed. While comparing the metal concentrations, 400 µgkg^−1^ of Cd had the maximum accumulation of CAT and POD in chickpea foliage. The build-up of antioxidant enzymes in the metal-treated plants was noticeably reduced when metal tolerant PGP strain PGPR-7 and T-4 strain was applied singly/combined to Cd-treated chickpea. For instance, when compared to uninoculated, plants inoculated with *P. fluorescence* PGPR-7 and treated with 25 µg Cdkg^−1^. Likewise, CAT activity was maximally reduced (55% reduction) when *Trichoderma* sp. T-4 was inoculated with 25 µg/kg of cadmium. Furthermore, dual inoculation of PGPR-7 and T-4 strains caused the maximum reduction of 29% and 32% in CAT and POD activity in 25 µg Cd kg^−1^ soil treated chickpea plants (Fig. 4). In conclusion, it is quite likely that metal tolerant microbial inoculants (*P. fluorescence* and *Trichoderma* sp.) lowered the amount of antioxidant enzymes, which would have otherwise increased in the presence of Cd stress. Even in soils with metal contamination, the overall growth of chickpea was significantly increased as a result of the clear decrease in antioxidants brought on by single/dual inoculation with the metal-tolerant microbes. Similar to our findings, it has been discovered that CAT activity was significantly increased in a concentration dependent manner in the leaf and root tissue of different legume seedlings^69^. Furthermore, Park et al.^70^ also noticed a decrease in oxidative stress (CAT and POD) of soybean plants following the inoculation PGPR *Bacillus aryabhattai* strain. Similarly, IAA producing and Cd tolerant *Burkholderia gladioli* and *P. aeruginosa* strains declined the level of antioxidant enzymes by lowering the Cd-uptake in tomato^71^. Likewise, phytohormone producing endophytic fungus *Penicillium roqueforti* decreased the metal induced oxidative stress in wheat and improved the growth^72^.

#### Proline and MDA content in inoculated and Cd-treated Chickpea

**Figure 5:**
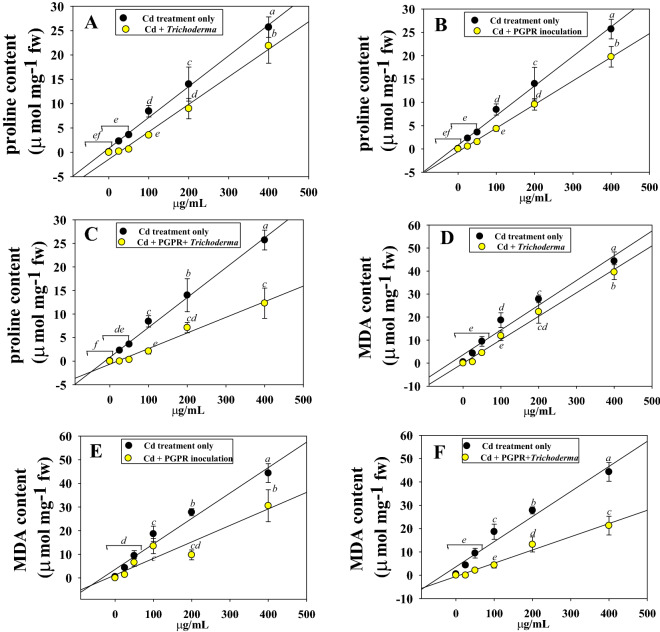
Proline content estimation in Cd treated chickpeas and inoculated with *Trichoderma* sp. T4 (**A**), PGPR-7 strain (**B**) and *Trichoderma* sp. T4 + PGPR-7 strain (**C**). Panels D, E and F represents the MDA content extracted from Cd-treated and inoculated with *Trichoderma* sp. T4 (**D**), PGPR-7 strain (**E**) and *Trichoderma* sp. T4 + PGPR-7 strain (**F**) chickpeas. Here, line diagram represents the mean of three replicates (*n* = 3). Corresponding error bars represent standard deviation (S. D.) of replicates. Different letters represent that mean values are significantly different according to DMRT test.

Proline is thought to act as an osmo-regulator, protecting plants from the harmful effects of ROS produced by plants when they are exposed to environmental challenges such heavy metals, salt and drought etc. Proline also helps plants maintaining homeostasis and osmotic equilibrium. In addition to this, it functions as a ROS scavenger and is essential for maintaining the subcellular structures of plants to help them survive a variety of environmental challenges. Taking this into account, the proline content of chickpea cultivated with or without Cd was measured. In general, when Cd level increases, the proline content in chickpea leaves gradually increased. It's interesting to note that when plants were cultivated in the presence of 400 µg Cd kg^−1^, compared to the uninoculated control, a 67% upsurge in proline concentration was observed. Conversely, following inoculation of metal tolerant microbial strains PGPR-7 and T-4, the accumulation of proline in chickpea foliage were significantly declined by 31% and 42%, respectively when plants were raised in soil polluted with 400 µg Cd kg^−1^ soil over uninoculated but treated with identical Cd doses (Fig. 5). The maximum decline (48%) in proline concentration was recorded when dual cultures were applied to chickpea plants grown in soil treated with 25 µg Cd kg^−1^ soil. A comparable decrease in proline content in the plant organs of Cd-treated *Hordeum vulgare* (L.) inoculated with mycorrhizal fungi^73^ and cultivated in stressful settings was observed, correlating with our findings.

Since, single/combined inoculation of metal tolerant microbial bacterial strain lessened the effects of Cd stress and shielded the plants from oxidative damage, the metal tolerant PGPR utilised in this study allowed chickpea plants to survive and flourish even under metal stress. These findings support the idea that metal tolerant microbial strains might function as a potential organism in bioremediation procedures, eventually improving the functionality of chickpea grown under metal stress.

**Figure 6:**
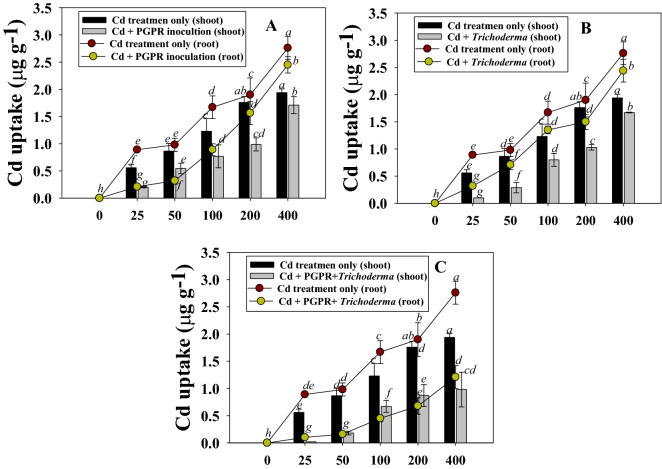
Uptake of Cd in chickpea plants treated with different concentrations** (**0, 25, 50, 100, 200 and 400 μg Cd kg^−1^) of Cd and inoculated with PGPR-7 (**A**), *Trichoderma* sp. T-4 (**B**) and PGPR-7 + *Trichoderma* sp. T-4 (**C**). Each bar and line diagram represents the mean of three replicates (*n* = 3). Corresponding error bars represent standard deviation (S. D.) of replicates. Different letters represent that mean values are significantly different according to DMRT test.

Many plants, including legumes, are affected by heavy metal stress that results in the production of ROS, which affects lipid peroxidation (LPO) in cellular membrane. Malondialdehyde (MDA) is a by-product of LPO in plant tissues. MDA thus serves as a marker of the intensity of stress for plants. In light of this, production of MDA was measured in chickpea leaves affected with varying dosages of Cd. With larger metal doses, leaves generally contained more MDA. For instance, compared to the un-inoculated control, 400 µg Cd kg^−1^ soil markedly raised the MDA concentration by 54%. However, even with 200 µg Cd kg^−1^ soil, *P*. *fluorescence* PGPR-7 and *Trichoderma* sp. T-4 were still effective in reducing metal stress and, consequently reduced the level of MDA by 43% and 49%, respectively (Fig. 6). Moreover, combined inoculation of bacteria and fungi had maximum detoxifying effect and lowered the MDA concentration maximally in chickpea plants contaminated with 25 µg Cd kg^−1^ soil. Saif and Khan^74^, investigated that HM stress-induced seedlings of chickpea had a significantly higher MDA and proline contents than control seedlings which however, decreased following the inoculation of Ni and Cr tolerant *Pseudomonas aeruginosa*.

#### Yield attributes and nutritional content in Cd-treated and bio-inoculated chickpeas

At harvest (120 DAS), the seed yield (SY) and seed protein (SP) showed a steady decline with successively increasing doses of Cd. However, the inoculation of metal tolerant microbial strain caused an increase in these parameters. The maximum number of seeds/plants (25 seed/plants) were recorded when chickpeas were detached from soil inoculated with microbial consortia (PGPR-7 strain and T4). Furthermore, chickpea plants inoculated with PGPR-7, *Trichoderma* sp. T-4 and PGPR-7 + T-4 and treated with 25 µg Cd kg^−1^ soil increased the seed yield by 26%, 28.5% and 32%, respectively over uninoculated but treated with similar Cd dose (Table 4). The seed protein content was also were significantly declined with increasing Cd concentration. In contrast, the Cd-tolerant *Pseudomonas* PGPR-7, *Trichoderma* sp. T4 and *Pseudomonas* PGPR-7 + *Trichoderma* sp. T4 on the other hand, enhanced the SP of chickpea by 20%, 27% and 38%, respectively, at 25 µg Cd kg^−1^ soil, when compared to uninoculated chickpea cultivated in soil amended with identical doses of cadmium. In a similar study, Oves et al.^75^ demonstrated *that Pseudomonas aeruginosa* improved the yield attributes of chickpea plants raised in chromium (Cr) contaminated soil. Furthermore, research by Tripathi et al.^76^ demonstrated that growth characteristics and yield of chickpea were improved when the plant was grown with a consortium of *Trichoderma* while being exposed to the arsenic (As). The statistical study significantly demonstrated how Cd concentrations and microbial inoculations both had an interaction effect on the examined parameter.

**Table 4:** Single and combined inoculation impact of metal tolerant *Pseudomonas fluorescence* PGPR-7 and *Trichoderma* sp. T-4 on yield attributes and nutritional content of chickpea raised in Cd-treated soils.

Treatment/ Bio-inoculants	Cd concentration (µg kg^−1^ soil)	Yield attributes			Nutrients	
		Number of seeds/plants	Seed yield (g plant^−1^)	Seed protein (mg g^−1^)	N content (mg/g)	P content (mg/g)
UC	0	14.0 ± 1.3	1.3 ± 0.02	213 ± 10.1	13.2 ± 1.2	2.3 ± 00
	25	13.0 ± 0.5	1.12 ± 0.1	200 ± 5.6	12.0 ± 1.8	1.71 ± 00
	50	12.0 ± 0.8	0.87 ± 0.08	176 ± 7.0	10.0 ± 0.0	1.34 ± 0.1
	100	10.0 ± 0.0	0.71 ± 0.03	143 ± 1.7	7.8 ± 0.7	1.0 ± 0.02
	200	8.0 ± 1.0	0.54 ± 0.01	123 ± 5.0	5.4 ± 0.3	0.56 ± 0.0
	400	2.0 ± 0.0	0.26 ± 0.0	78.4 ± 11.0	2.2 ± 0.0	0.15 ± 0.01
Mean	**–**	9.8	0.8	155.5	8.4	1.17
*P. fluorescence* PGPR-7	0	18.0 ± 2.3	1.76 ± 0.1	267 ± 23.0	16.7 ± 0.1	2.67 ± 0.2
	25	16.0 ± 1.8	1.5 ± 0.03	252 ± 8.9	14.3 ± 2.4	2.55 ± 0.4
	50	14.0 ± 2.0	1.32 ± 0.09	233 ± 0.0	12.4 ± 3.1	1.82 ± 0.6
	100	12.0 ± 1.1	1.1 ± 0.01	222 ± 7.7	10.2 ± 0.8	1.34 ± 0.0
	200	10.0 ± 1.0	0.83 ± 0.03	145 ± 8.1	8.2 ± 0.5	1.10 ± 0.0
	400	5.0 ± 0.0	0.54 ± 0.03	110 ± 5.9	6.4 ± 0.7	0.76 ± 0.01
Mean	**–**	10.8	1.17	204.3	11.3	1.70
*Trichoderma* sp. T-4	0	20.0 ± 3.1	1.82 ± 0.02	292 ± 11.0	15.0 ± 2.1	2.44 ± 0.6
	25	18.0 ± 0.8	1.71 ± 0.1	267 ± 5.4	14.0 ± 1.6	1.98 ± 0.3
	50	15.0 ± 2.8	1.43 ± 0.04	255 ± 4.8	13.1 ± 2.0	1.56 ± 0.4
	100	13.0 ± 1.5	1.31 ± 0.0	231 ± 6.0	12.0 ± 0.0	1.44 ± 0.0
	200	11.0 ± 0.0	1.12 ± 0.0	178 ± 5.0	11.0 ± 1.1	1.21 ± 0.02
	400	6.0 ± 0.2	0.76 ± 0.0	165 ± 0.0	8.1 ± 0.0	0.92 ± 0.01
Mean	–	13.8	1.35	231.3	12.2	1.59
*P. fluorescence* PGPR-7 + *Trichoderma* sp. T-4	0	25.0 ± 1.7	1.91 ± 0.01	341 ± 4.5	21.3 ± 2.4	2.89 ± 0.6
	25	23.5 ± 0.9	1.78 ± 0.2	289 ± 6.6	18.9 ± 3.0	2.45 ± 0.2
	50	22.0 ± 0.4	1.56 ± 0.3	276 ± 3.8	17.2 ± 1.2	2.22 ± 0.1
	100	20.0 ± 0.8	1.42 ± 0.05	245 ± 7.0	15.6 ± 0.8	1.89 ± 0.0
	200	16.0 ± 1.2	1.32 ± 0.0	211 ± 0.0	13.2 ± 0.6	1.56 ± 0.1
	400	12.0 ± 0.0	1.0 ± 0.01	187 ± 6.5	11.4 ± 0.3	1.21 ± 0.0
Mean	–	19.6	1.50	258.1	16.2	2.03

Each value is a mean of three replicates where each replicate constituted three plants/pot. Mean values are significantly at *P* ≤ 0.05. Means followed by similar alphabets are different from each other according to DMRT test. Here, Cd = cadmium, µg/kg = micrograms per kilograms, N = nitrogen, P = phosphorous.

Understanding the toxic effect of heavy metals on nutritional values of legumes and the efficiency of Cd-tolerant microbial consortia in toxicity removal and enhanced uptake of nutrient was evaluated. Here, the increasing Cd doses declined the nitrogen and phosphorous content of chickpea. However, inoculation of *Pseudomonas* PGPR-7, *Trichoderma* T-4 and *Pseudomonas* PGPR-7 + *Trichoderma* T-4 significantly increased the N (21, 13 and 39%, respectively) and P (14, 6 and 20%, respectively) content at 25 µg Cd kg^−1^ soil (Table 4). Two elements for the computed components, an ANOVA revealed that the single and dual effect of microbial inoculants and their interaction (inoculation + cadmium) were significant (*p* < 0.05). In almost treatment, the dual inoculation of microbial consortia potentially mitigated the hazard of Cd and maximally increased/improved the biometric parameters.

#### Cd uptake in inoculated chickpeas

Although there were significant differences in the amount of Cd uptake in roots and shoot tissues of chickpea, the concentration of metals consistently rose with higher Cd dosages. In terms of plant organs, shoots (1.89 µg g^−1^) and roots (2.78 µg g^−1^) exhibited the highest Cd concentrations when detached from soil contaminated with 400 µg Cd kg^−1^. Intriguingly, the plant organs showed the greatest decrease in metal concentration after inoculation of Cd-tolerant *P. fluorescence* PGPR-7 and *Trichoderma* sp. T4. For instance, the Cd uptake by roots that were removed from plants growing at 400 mgCdkg^−1^ was reduced by 43% after inoculation of *P. fluorescence* PGPR-7 strain (Fig. 6). Similar to this, it was found that *Trichoderma* sp. T4 inoculated plant roots had a 37% decrease in metal deposition in the presence of 400 µg Cd kg^−1^. The reduction/decrease in Cd uptake following metal tolerant microbes was observed followed the order: *P. fluorescence* PGPR-7 + *Trichoderma* sp. T4 (dual inoculation) > *Trichoderma* sp. T4 > *P. fluorescence* PGPR-7. Although cadmium (Cd) is a highly hazardous element, Cd ions are easily transferred from the soil to different plant components. It's possible that this is caused by the production of protons, root exudates, and other metabolites, which facilitate the simple dissolution of Cd ions, most likely by way of the formation of metal chelating complexes. Therefore, plants inoculated with metal tolerant microbial species could showed the decreased metal deposition in each organ, probably as a result of the metal-tolerant strain accumulating the majority of the metal component present in the soil. Because of this, the plants could only absorb a lesser amount of metal. Thus, when metal pressure was released, the inoculated chickpea plants had a better chance of surviving even in soil that had been treated with Cd. In a similar manner, Cd-tolerant strain *Bradyrhizobium* lowered the uptake of cadmium in *Glycine max* (L.) and *Lolium multiflorum* (L.) by reducing the toxicity^77^. Furthermore, metal tolerant *T*. *harzianum* increased the growth of *Hordeum vulgare* (L.) by decreasing the uptake of Cd^78^.

## Conclusions

The present investigation was primarily concerned with the detrimental effects of Cd on biological and chemical properties of chickpea. It was observed that Cd-induced severe oxidative damage in chickpeas led to membrane LPO and a powerful antioxidant defence response. Either singly/combined inoculation of metal-resistant *P. fluorescence* PGPR-7 and *Trichoderma* sp. T-4 prevented Cd toxicity in chickpeas. Even under Cd pressure, microbial strains were remarkably capable of producing a variety of growth regulating active biomolecules. These elements may have contributed significantly to improving the functioning of chickpeas, either alone or concurrently, and they have shed light on why chickpea plants kept growing and functioning even when exposed to Cd stress. Consequently, it was discovered that both microbial strains were effective/potential candidates that could be used to remediate the metal contaminated soils. As a result, the performance of Cd-treated chickpeas as a whole was improved when combined inoculum of both strains was used. The Cd-resistant *Pseudomonas fluorescence* and *Trichoderma* sp. could, therefore, serve as a potent microbial inoculant that could be used in a straightforward, affordable, and environmentally friendly method to remediate the heavy metal contaminated soils because of the commendable metal tolerance ability and other distinctive growth promoting features. Furthermore, from the study, it is recommended that irrigation of edible crops with industrially discharged effluents/wastewater should be minimized. In a nutshell, we can say that the applied HM-tolerant microbes could play an essential in agricultural sectors as an army to clean-up/decontaminate the heavy metal hazard. Furthermore, efficacy of HMs tolerant microbes can be increased by transferring the genes from theses HM-resistant microorganisms to another laboratory cultivated genetically engineered microbes.

## Data Availability

Sequence data of identified bacterial strain used in this study have been submitted to the NCBI archives (https://ncbi.nlm.nih.gov). The name of the repository and accession number can be found here; https://www.ncbi.nlm.nih.gov/genbank/, OP925885.
